# *Staphylococcus aureus* Dissemination Presenting With Encephalopathy and Epidural Abscess

**DOI:** 10.1155/crdi/6889110

**Published:** 2024-11-18

**Authors:** Dhriti Sundar Das, Anupam Dey, Gurudip Das, Suprava Naik

**Affiliations:** ^1^Department of General Medicine, All India Institute of Medical Sciences (AIIMS), Bhubaneswar, India; ^2^Department of Orthopedics, All India Institute of Medical Sciences (AIIMS), Bhubaneswar, India; ^3^Department of Radiodiagnosis, All India Institute of Medical Sciences (AIIMS), Bhubaneswar, India

**Keywords:** bacteremia, encephalopathy, epidural abscess, pneumonia, sepsis, *Staphylococcus aureus*

## Abstract

**Background:** Staphylococcal infection is a common bacterial disease with common clinical features. Untreated infection, especially in immunosenescence cases, can affect other organs. This can lead to multiorgan dysfunction and cause increased morbidity and mortality. Unlike commonly presented features of pneumonia, dissemination of infection can pose diagnostic and therapeutic enigma. Therefore, any such presentation in common clinical practice can yield a conundrum of diagnoses.

**Case Report:** A 69-year-old elderly male presented to the Emergency Department with acute onset encephalopathy. Historically, cues were limited, and evaluation was negated for acute cerebrovascular event or seizure. Laboratory findings were suggestive of a severe sepsis. While clinical medicine workup and diagnostic dilemma were ongoing, possible sources of the sepsis were thoroughly sought including range of infectious causes. This patient's presentation was one of its kind: staphylococcal bacteremia seeding to cause pneumonia and unusual epidural abscess in due course of illness.

**Conclusion:** The health outcome of the critically ill especially elderly patients depends mostly on the importance of clinical medicine to address the diagnostic enigma and virtue of supportive care delivered. *Staphylococcus aureus* infections are capable of developing distant infectious foci, as highlighted in this case, and that the clinician should be alert to this possibility. This particular case firmly posits an admonition for clinicians and the importance of clinical medicine for critical reasoning to improve the patient outcome.

## 1. Background

Staphylococcal infections may range from skin infections to life threatening conditions, namely, abscess formation, sepsis, pneumonia, and cardiovascular infections [1]. Spinal epidural abscess is an uncommon but significant cause of low back pain in the critically ill patients [2]. Over the decades, genetic and molecular analysis of *Staphylococcus aureus* revealed various adhesion substances, superantigens and evasion of immune mechanism that explains distant infections and invasion [3]. Population studies have persistently revealed that young and elderly male individuals are at heightened risk of these bacterial infections [1]. Uncontrolled diabetes can lead to infection by other uncommon microorganism [4]. Extensive epidural abscess have also been reported in elderly subjects with the mentioned risk factors [5]. Community acquired pneumonia (CAP) requiring hospitalization amounts to 2 to 5% of staphylococcal infections [6]. *Staphylococcus* causing empyema occurs commonly and is responsible for about 20% of cases [7]. In community settings, *S. aureus* is the second most common cause of bacteremia [8]. Moreover, such bacteremia are usually associated with identifiable sources such as pneumonia, underlying abscess, or joint infections [9]. Pyogenic abscess of the epidural region demands urgent management to prevent serious neurologic deterioration or death. Management of *Staphylococcus* blood stream infections (BSI) requires prompt antibiotic administration without delay and identification of the source and removal of the same [10]. This particular case report highlights a complex unique critical patient with *Staphylococcus* BSI that manifested as pneumonia with epidural abscess. The objective of this case is to put forward the importance of clinical medicine to rapidly identify and manage such critically ill which has bearing on the future patient outcome.

## 2. Case Report

This was a case of 69-year-old male presented to the hospital with the diagnosis of right lower lobe pneumonia with a sepsis with methicillin sensitive *S. aureus* bacteremia. He was normal 7 days prior to admission when he developed acute onset altered sensorium. He was a known diabetic on oral medications. There was no reported history of fever, cough, or rashes anywhere in the body.

On examination, the patient was hemodynamically stable and had no signs of meningeal irritation. However, he was found to have decreased infrascapular breath sounds. Cardiac auscultation revealed no murmurs.

**Figure 1:**
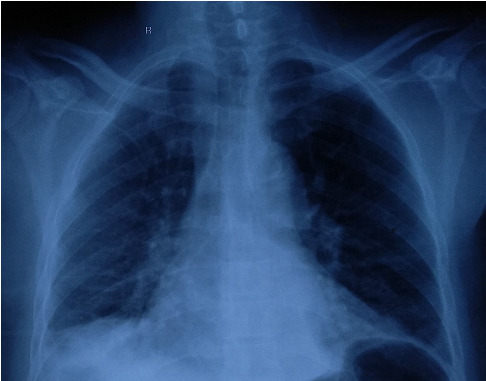
Chest X-ray showing right-sided CP angle blunted.

**Figure 2:**
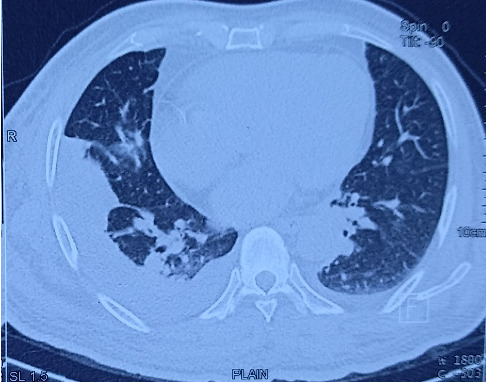
High-resolution CT of the chest showing right lower lobe consolidation and pleural effusion.

Laboratory evaluation were significant for leucocytosis (leucocytes, 23 × 10^9^/L; reference range, 4.0–11 × 10^9^/L) with neutrophilic predominance of 93%, hyponatremia (sodium, 124 mEq/L; reference range, 135–145 mEq/L), lactate of 1 mmol/L (reference range, < 2 mmol/L), and HCO_3_ 19.6. His random blood sugar on presentation was 263 mg/dL. ESR was 47 mm at presentation which decreased to 43 mm during the course of the illness. However, C-reactive protein was not done due to unavailability. Chest radiograph showed blunting of right costophrenic angle suggestive of pleural effusion (Figure 1). Echocardiography was normal. High resolution computed tomogram of the chest revealed right lower lobe consolidation with pleural effusion (Figure 2). Blood culture was sent to the microbiology lab of the academic center which showed methicillin-sensitive *S. aureus* (MSSA) positive. Noncontrast computed tomogram (NCCT) of the brain was normal.

The patient was promptly started with intravenous broad spectrum antibiotics, namely, injection piperacillin tazobactam in the dose of 4.5 gm every 6 h and intravenous fluids and other supportive measures. After 48 h of admission, he developed acute onset weakness of both the lower limbs. It was accompanied by retention of urine.

**Figure 3:**
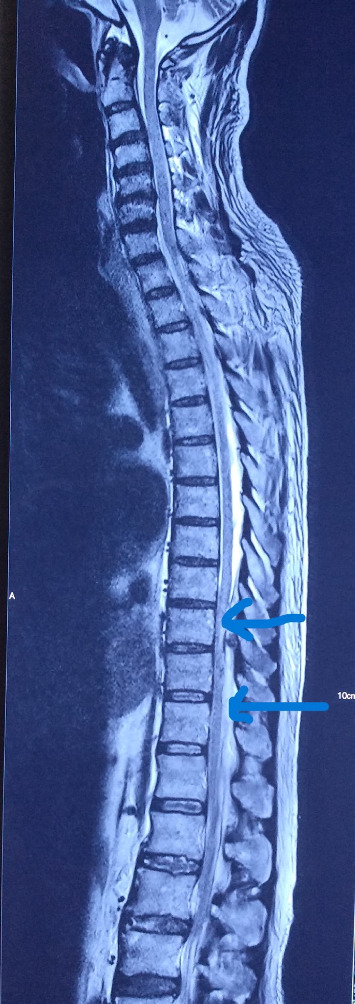
Magnetic resonance imaging of the spine showing T8-L1 spinal epidural abscess in the T2 weighted image (sagittal view).

**Figure 4:**
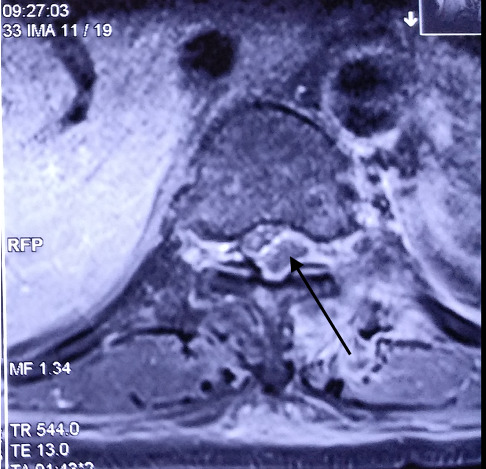
Magnetic resonance imaging of the spine showing T8-L1 spinal epidural abscess in the T2 weighted image (axial view).

**Figure 5:**
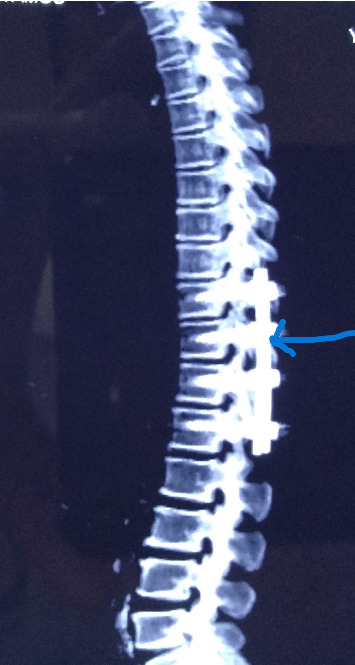
NCCT spine (post drainage and fixation).

An urgent MRI spine was done which revealed an epidural lentiform-shaped collection extending from T8 to L1 in the T2 weighted image which was consistent with abscess (Figures 3 and 4). As he developed neurological deficits, urgent intervention was indicated. The patient underwent emergency neurosurgical intervention with drainage of the abscess and fixation (Figure 5). Around 25 mL of pus was aspirated from the epidural space and sent for culture and sensitivity which was positive for the same organism MSSA. The patient responded to the treatment with improvement of the sensorium and his neurological deficit.

## 3. Discussion

This report highlights the importance of outlook in clinical medicine in managing common infection with unusual presentation. Although treating physicians are confronted with deluge of clinical data but identifying the crucial one in that critical juncture holds the key to good outcome. The back pain in the setting of the hospitalized patient may be easily overlooked or ignored attributing to trivial reasons. More so when such a patient is critically ill with the sepsis and have pneumonia. The initial presentation of acute onset of encephalopathy seems to be due to the underlying sepsis and pneumonia. These manifestations continued to improve with antibiotics, intravenous fluids, and other supportive care.

Despite the initial presentation of encephalopathy and the improvement, he was brewing metastatic infection in the form of epidural abscess. Due to the encephalopathy, the patient was unable to report the history of low back pain. Thereafter, the abscess may enlarge enough to cause neurological deficits in the form of paraparesis with autonomic involvement. In severe cases of *Staphylococcus* bacteremia, distant infection in the form of deep-seated abscess may occur which require removal for optimal resolution of the infection [9, 10]. The pooled prevalence of methicillin-resistant *S. aureus* in this part of Eastern India is 49% [11]. It was around 41 to 42% in 2008 to 2009 [12]. While the sepsis with septic encephalopathy may be the initial cause of his presentation, but development of new onset weakness of both lower limbs during the in-hospital course warrants immediate intervention. Spinal epidural abscess revealed on MRI indicates the seriousness of the course of illness [13].

Of note, earlier case report published on spinal epidural abscess reported to have chronic low back pain which on further course of illness found to have the condition [2]. However, in our case, due to the altered level of sensorium in the patient, he was unable to give proper history. Only when he developed neurological deficits, the whole clinical picture came to the limelight. Therefore, this case is not only unique in the setting of acute encephalopathy with revealing pneumonia and sepsis but also the onset of new neurological deficit adds to its uniqueness. The initial presentation of altered sensorium widens the differential diagnosis ignoring possibilities of deep-seated abscess within. While it might be possible that the patient might have back pain for quite some time which he might have ignored, the initial clinical presentation of the case typically deviates one from suspecting underlying serious metastatic infections.

## 4. Conclusion

High index of clinical suspicion is of utmost importance in managing geriatric patients with comorbidities presenting with acute onset encephalopathy. Vigilant work-up and prompt action form the key in arriving at an early diagnosis in these patients. *S. aureus* infections are capable of developing distant infectious foci, as highlighted in this case, and that the clinician should be alert to this possibility. Due diligence needs to be ensured to improve the heath outcome in these multimorbid patients. The paramount importance of clinical medicine is being highlighted in this case report.

### 4.1. Importance of the Case Report

This case stresses the importance of extreme vigilance and prompt intervention in managing spinal epidural abscess when the same is high in the differentials.

## Data Availability

The data regarding the case report are available from the corresponding author and the same may be obtained on request after obtaining appropriate institution approval.
